# Supporting Minimally Verbal Autistic Girls with Intellectual Disabilities Through Puberty: Perspectives of Parents and Educators

**DOI:** 10.1007/s10803-018-3782-8

**Published:** 2018-10-24

**Authors:** Clare Cummins, Elizabeth Pellicano, Laura Crane

**Affiliations:** 1 Centre for Research in Autism and Education (CRAE), UCL Institute of Education, University College London, London, WC1H 0NU UK; 2 Macquarie University, Sydney, Australia; 3 Present Address: Division of Psychology and Language Sciences, University College London, London, UK

**Keywords:** Autism, Puberty, Minimally verbal, Intellectual disability, Parents, Teachers

## Abstract

Limited research has examined puberty in autistic girls, yet alone those who are minimally verbal and with additional intellectual disabilities. In this study, ten parents and ten educators were interviewed about their views and experiences of supporting these girls through puberty. Results demonstrated that many parents had concerns prior to the onset of puberty in these girls. Yet, for most girls, experiences of puberty were felt to be positive, with the girls coping well with changes that they were experiencing (e.g. menstruation, breast development and developing body hair). Thematic analysis of interview data highlighted three main themes: a range of individual experiences and needs; the importance of promoting dignity and respect; and identifying ways to support these girls through puberty.

Puberty is a time of immense change in a person’s life, relating to the physical changes that occur as part of the transition from childhood to adulthood (Grover and Bajpai 2008). For girls, this transition typically begins around 11 years of age, and occurs in a sequence of accelerated growth, breast development, adrenarche (resulting in production of pubic hair, body odour, and skin oiliness), and menarche (the first occurrence of menstruation) (Raine et al. 2011). These physical changes combined with simultaneously occurring psychosocial, emotional and behavioural changes, comprise what is known as adolescence. While this time brings marked changes, little research exists on the experience of puberty for autistic^1^ girls, and even less for those who are minimally verbal and with additional intellectual disabilities.

Research in girls with intellectual disabilities highlights a range of potential challenges, and possible positive outcomes, associated with puberty. Generic issues associated with puberty (such as mood changes and period pains) do arise (Albanese and Hooper 2007) and these may be particularly pertinent for girls with limited spoken communication (as they are often only detected through increased agitation or upset, and parents may not be aware of the cause of such behaviours). Physical changes may also pose challenges: growth related to puberty may cause difficulties for girls who require physical assistance for mobility, and breast development may cause discomfort, especially for girls who wear harnesses on transport (Zacharin 2009). Menstrual management may also be particularly challenging, with women with intellectual disabilities reporting menstruation to have a negative impact on their well-being (Ditchfield and Burns 2004), and caregivers often favouring medical management of menstruation (Carlson and Wilson 1994). To minimise these challenges, it has been suggested that special educators should be involved from the onset of menstruation, with teaching customised to each girl’s level of understanding and independence (Albanese and Hooper 2007; Joshi and Joshi 2015). Whilst parents express fears relating to the onset of puberty in this population (e.g., unwanted sexual exposure, contraceptive needs, loss of childhood), these fears are rarely actualized (Zacharin 2009). Potential benefits of people with severe intellectual disabilities reaching puberty have also been noted. These include sex hormones potentially increasing physical and psychological maturity. According to parental report, these appear to result in increased physical independence and improved mood (Zacharin 2009).

Research on puberty in autistic girls specifically has tended to focus on menstrual management. A recent survey by Steward et al. (2018) found that autistic people report increased sensory, emotional and behavioural difficulties in relation to menarche. Whilst these issues are also found in non-autistic people, autistic people felt it placed an extra strain on their lives. Menstruation is also an area of concern for parents of autistic girls (Cridland et al. 2014), with some parents seeking medical intervention to suppress or, in extreme circumstances, eliminate it (Koller 2000; Memarian and Mehrpisheh 2015). However, these same parents often express surprise by how well menstruation has been managed by their daughters (Koller 2000; Memarian and Mehrpisheh 2015).

An important aspect of menstrual management is education, with Koller (2000) highlighting the need for teaching to be adapted to the needs of each learner (an idea also advocated for by post-menarcheal autistic adults; Steward et al. 2018). Potential approaches to teaching menstrual management include: ‘chaining’ (i.e. breaking a skill down into a number of smaller units and having the successful acquisition of each small step reinforced) (Veazey et al. 2016), and the use of Social Stories (short individualised descriptions of specific situations, written in a specific style and format, explaining what is expected in that situation and why; Gray 2010) (Klett and Turan 2012). It has also been suggested that girls should be prepared for menarche early in development, by teaching pre-requisite skills such as toileting, dressing, and following instructions in daily routines (Memarian and Mehrpisheh 2015; Veazey et al. 2016).

Despite emerging research on puberty in girls with intellectual disabilities, as well as puberty in autistic girls, limited research exists on this topic in autistic girls who are minimally verbal. There is no universally accepted definition of ‘minimally verbal’, yet Tager-Flusberg and Kasari (2013) use the term to encompass those with no spoken language, a small repertoire of expressive vocabulary, or those with primarily scripted language or limited fixed phrases that are used communicatively. It is estimated that appropriately 30% of the autistic population can be classified as minimally verbal (Tager-Flusberg and Kasari 2013). As with the general autistic population, autistic individuals who are minimally verbal are a heterogeneous group who vary in terms of intellectual disabilities (Munson et al. 2008). Intellectual disabilities are categorised by challenges regarding both intellectual and adaptive functioning, across social, cognitive, and practical domains (DSM-5; American Psychiatric Association 2013).

The aim of the current study was to explore the experience of puberty for minimally verbal autistic girls with intellectual disabilities through conducting semi-structured interviews with a sample of the girls’ parents and educators. By better understanding the experience of puberty in this population of girls, this preliminary study aimed to identify any concerns prior to the onset of puberty in these girls, and to determine the most effective ways of supporting the girls (and their parents and educators) through this crucial developmental stage.

## Method

### Participants

#### Parents

Ten parents (nine mothers and one father) of nine minimally verbal autistic girls with intellectual disabilities, all of whom were currently experiencing puberty, participated in the research. Parents were all based in the United Kingdom and Ireland, and their daughters were, on average, 14 years and 8 months of age (range 11 years 4 months to 19 years 11 months, SD = 11.91 months). Their daughters were all attending special schools and were included in the study based on parental confirmation of inclusion criteria (i.e., that they had a formal diagnosis of an autism spectrum disorder and also intellectual disability, and met the Tager-Flusberg and Kasari (2013) definition of being minimally verbal). When questioned about the aspects of puberty their daughters had experienced, all nine girls were reported to have started their periods, with eight said to have experienced period pains. Six girls were developing breasts, four were developing body hair, five were developing spots, and three were reported as having to bathe more often.

#### Educators

Ten educators, all of whom had worked in an educational capacity with the target sample (i.e., minimally verbal autistic girls with intellectual disabilities who had experienced puberty) took part. All educators were female and had worked with this population of girls for an average of 3 years and 5 months (range 3 months to 10 years, SD = 10.76 months). The sample comprised seven teachers, one tutor (working with students on a 1:1 basis), and two supervisors (who created and oversaw the implementation of students’ individualised education plans). All educators worked in special schools in the United Kingdom and Ireland.

### Materials

Two interview schedules were created for this study: one for parents and one for educators. Both interview schedules followed a semi-structured format, consisting of four primary (open) questions, and a number of related prompt questions (used to probe for further details, where appropriate) (see Appendix for details).

### Procedure

Ethical approval was granted via the Department of Psychology and Human Development at UCL Institute of Education, University College London.

Parent interviews lasted an average of 20 min (range 6–50 min). Four interviews took place in person (with two parents being interviewed together), four took place over the phone and one via Skype (according to participant preferences and/or feasibility). Educator interviews lasted an average of 22 min (range 12–34 min) with five conducted in person, and five via phone call. All interviews were audio recorded and transcribed verbatim.

Interview data were analysed using thematic analysis (Braun and Clarke 2006). An inductive approach was used, meaning that identified themes did not fit into pre-existing frameworks and no attempts were made to engage with previous literature to enhance the analysis. Data were analysed at a semantic level, with themes identified from the surface meaning of the data. The process of analysis involved: transcribing; actively reading and re-reading transcripts of interviews and generating initial notes; manually coding the data set; and sorting the codes into potential themes and subthemes. The potential themes and subthemes were refined using an iterative process (both in relation to the individual codes and the entire data set), before naming, defining and reporting on themes. While there were clear stages to the analyses, these were not followed in a linear fashion; it was necessary to move backward and forward within the process of data analysis. Further, to ensure reliability, two of the authors (CC and LC) independently analysed the data (with oversight from EP), and then met several times to discuss potential themes before deciding on the final themes and sub-themes.

## Results

**Fig. 1:**
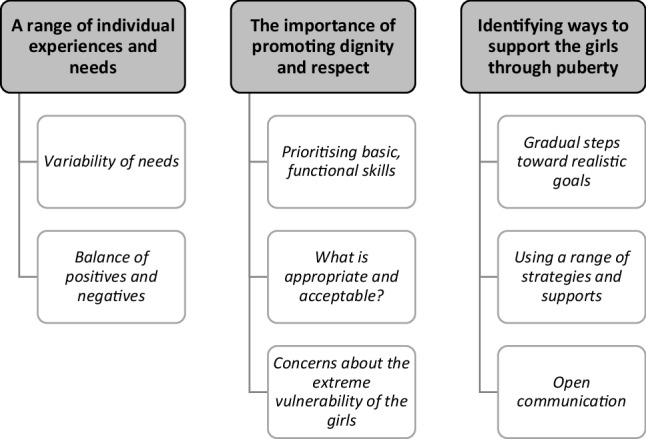
Themes and sub-themes identified from the interviews with parents and educators

Three themes were identified from the parent and educator interviews: (1) a range of individual experiences and needs; (2) the importance of promoting dignity and respect; and (3) identifying ways to support the girls through puberty. Themes and sub-themes are presented in Fig. 1 (sub-themes are also presented in italics).

### Theme 1: A Range of Individual Experiences and Needs

Parents and educators highlighted how minimally verbal autistic girls with intellectual disabilities were experiencing similar pubertal changes to other, typically developing girls (e.g., menstruation, breasts developing, body hair developing). However, there was a clear *variability of needs* with regards to each individual girls’ ability to manage and cope with these changes. For example, all parents talked about the girls learning to manage their menstrual hygiene, but each was managing this to varying extents. Whilst some girls were confident and independent: “She puts her pad on herself, she takes it off herself and puts it in the bin” (parent); others required much more support: “I think she’s still at the stage of being shown the pad and how to put it in…. and wiping” (parent). For many girls, this was related to their general level of skill and ability, irrespective of their ages: “she hasn’t got any of the motor skills or the awareness… you know, tampons for typical women can be a hassle” (parent).

Interviewees reported that some of the girls were unfazed by the experience of puberty, yet others struggled. These struggles manifested in different ways, with some girls demonstrating specific behaviours: “she does suffer horrendous pre-menstrual tension (PMT) and she does have a lot of behaviours a week before her period” (parent); and others becoming more subdued or “teary” (parent). Parents and educators highlighted how it was critical to be astute to subtle changes in behaviour (e.g., girls touching their stomachs, having increased appetites), and—if appropriate—making changes to accommodate the girls’ needs: “because they weren’t able to verbalize their pain, you have to kinda just watch them… just predict what the pain level is, and what they need” (educator). Some parents and educators talked about managing this with a contraceptive pill, pain killers, reducing demands, or using hot water bottles: “Parents would give them pain relief at home if they thought they had it [their period], or they would come into school to give it to them because we actually weren’t allowed” (educator).

Teaching puberty-related skills was also reported to be a challenge, given the girls’ varying levels of understanding and awareness of puberty. This sometimes led to parental frustration, as the resources available to them did not always meet their daughters’ needs. For example, while some parents and educators praised the use of Social Stories in supporting the girls through puberty: “that worked quite well, to explain that, for example, you have to leave your pad on, you change it only in the bathroom” (educator); others found them inaccessible: “what happens when you can’t use Social Stories? That’s what people need to ask themselves. Because there’s a lot of girls who can’t… not meaningfully” (parent). Likewise, educators found whole-class teaching of puberty-related issues to be challenging, as it was difficult to meet the needs of every girl: “my advanced group, they’re learning how to differentiate between a stranger and a known person, and my less advanced group, the earliest of the earliest, is learning how to look at one of her classmates. So when I do group [work], I need to consider that” (educator).

Both parents and educators had mixed feelings about the changes that occurred during puberty for the girls, highlighting a *balance of positives and negatives*. Most parents were satisfied with how their daughters’ bodies were developing (e.g., developing breasts, getting their periods) and their daughters seemed to respond positively to these changes: “she likes to think of herself as a big girl. If you buy her a new stretchy bra thing that is marked as sort of sports thing, she quite likes to wear it” (parent). However, concern was expressed with regards to period pain, particularly for girls who could not express themselves verbally except using “signs of distress”. Parents and educators also reported sadness over the fact that these girls could miss out on favoured activities when menstruating, and they could not comprehend the reasons for this: “they’re not getting to do the things they want to do like swimming or trampolining that we go to once a week because they can’t really participate as well, because they’re bloated, they’re uncomfortable and they probably don’t understand why” (educator).

Notably, one parent felt her daughter’s autism served as an advantage in terms of her experience of puberty. While her intellectual disabilities and limited motor skills made learning new tasks difficult, her parents attributed her ability to learn these new routine skills to her autism: “In some ways it might be her best skill, because you know she is quite rigid in what she learns. If you can teach her exactly what you do, in an exact kind of situation, she will be able to do it” (parent). Another positive change noted by a parent was that her daughter learnt to speak upon reaching puberty: “she wasn’t speaking at all before puberty, and she started talking because of her puberty… learning-wise it was like a really big leap for her” (parent).

### Theme 2: the Importance of Promoting Dignity and Respect

When learning puberty-related skills, parents and educators placed an emphasis on *prioritising basic, functional skills*. Whilst some skills were deemed to be “good to have” (e.g., putting on deodorant, shaving), these were felt to be of lesser importance than more intimate skills (e.g., menstrual care): “coming from a point of view of dignity and independence I think the more intimate the skill, the more important it is to work on” (educator). A priority set for most girls was managing their own menstrual care (i.e., putting on and taking off a pad) and the ultimate goal was to become as independent as possible in completing these intimate tasks. For some, this process could be lengthy: “I’m confident by the time she’s 19 [in 8 years], she’ll be pretty good. I mean I don’t know how fully independent she’ll be, but I’m confident she’ll be able to, with her watch [a vibrating watch, set to vibrate at set intervals, to remind the daughter to go to the toilet to change her pad], take herself to the loo and change her pad” (parent). It may also require high levels of support: “we open everything out for her and then we just sort of say right pad off, you know, into bag, pad on, so it’s very minimal language and minimal steps to keep it really simple” (parent). Perseverance was felt to be key: “[another caregiver] was putting on like five pairs of underwear [rather than using sanitary pads] and I said you can’t do that, you know. She has to learn…. and now, a couple of years later… sometimes I wouldn’t even know she’s got [her period] because I could go into the bathroom in the morning and I’d see the papers in the bin and I’d go ‘oh, she must have got her period’. She just goes now and does it herself” (parent).

Throughout the interviews, parents and educators raised questions regarding *what is appropriate and acceptable* when teaching skills, managing behaviour and talking about puberty. In several interviews, discussions of appropriateness were raised in relation to masturbation. For some, a key task was to teach girls about the appropriateness of engaging in these activities privately, as opposed to publicly: “she mainly does it in the bath… so I always say to her that’s fine, that’s a really good place to do that, cause it’s in the bath and it’s private and other people are not here, and keep it very simple, and she seems to think that’s fine” (parent). Encouragingly, some girls responded well to such direction: “I took her up to her room and said ‘this is where you go’ and after that she used to just go off upstairs to her room on her own, close the door” (parent).

Whilst it was agreed that a key skill for girls to learn was “to be independent in the toilet and private when masturbating” (educator), there were conflicting views on how this should be addressed. First, interviewees expressed differing opinions regarding when the teaching of intimate skills should begin. Some felt it was important to start teaching puberty-related skills early on, as it generally took the girls a long time to acquire new skills: “Maybe consider teaching them before they start going through stuff… it would be nice for them to have a grasp of that already before it’s happened” (educator). Examples of how this could be achieved included using food colouring on underwear and practicing changing or wearing sanitary pads prior to puberty, to get accustomed to having to do so when necessary. Conversely, others felt that menstruation-related skills should only be taught when girls had begun menstruating, so they were more likely to know when to use these skills in specific situations: “it was more beneficial to do skills related to literally putting in sanitary towels and stuff like that when you are actually on your period. Because otherwise you don’t learn when you need to do it” (educator). There was, however, a consensus that basic, related skills (e.g., wiping oneself, developing the fine motor skills needed for changing a sanitary pad) should be focused on as early as possible: “in terms of gross motor movement, fine motor movement, trying to build that up before you teach them how to put a pad on [is helpful]” (educator).

Interviewees also expressed differing views in relation to the role of educators with regards to masturbation. One educator used Social Stories in response to girls engaging in these behaviours, while another felt it was inappropriate for educators to be overly involved in this aspect of puberty. One educator did mention experiences whereby parents were not very “reachable or accepting of what was happening”, and they had to address this by teaching girls to go to the toilet and do this privately: “because it was more of a private space… that was the most appropriate we could get it from her masturbating in the classroom” (educator).

Parents also highlighted the importance of appropriate language when discussing puberty: “people really still don’t have the language to know how to describe female anatomy or what’s happening, whether it’s periods or menstruation, people are very embarrassed and they’re still in the dark ages” (parent). For some parents, this was especially important when speaking directly to the girls: “[we ensure that my daughter’s] periods are referred to in a certain way… [we say] that her pants are ‘messy’, because ‘dirty’ gives the wrong connotation. Because it’s not a dirty thing to have a period, it’s just messy” (parent).

Both parents and educators shared *concerns about the extreme vulnerability of the girls;* the less independent the girls were, the more vulnerable they became. Even if the girls were independently managing their own toileting needs, they may still be highly dependent in managing their menstrual care: “it’s ok for us to deal with all aspects, but having reliance on other people is a bit of a worry for me” (parent). Increased dependency on others also increased the girls vulnerability as their experience of puberty became more public: “she’ll have her period and it’s going to be out there, in the whole class… so it’s quite a public experience and sometimes I feel quite protective and sad about that for her because she won’t ever be able to keep it secret, I mean not that it should be a secret but, you know, for your own sort of dignity, you don’t want everyone knowing you’ve got your period” (parent). Both parents and educators highlighted the relationship between the girls developing physically and them becoming more vulnerable: “I would have preferred if she wasn’t as developed like now because she doesn’t have the understanding, so the problem is she’s more vulnerable” (parent). In response to this increasing level of vulnerability, educators promoted strategies such as teaching the girls to say no to things they did not want, explicitly teaching what is private/public, and explaining what is inappropriate: “you know verbally ‘you do not’ you know, ‘don’t let anyone touch you’, ‘don’t do that’ or ‘if somebody touches you, you have to speak to someone’” (educator).

### Theme 3: Identifying Ways to Support the Girls Through Puberty

While some girls were successful in independently managing their menstrual care, parents reported that the majority of the girls were working on skills over a longer period of time, taking *gradual steps toward realistic goals*. In some cases, this learning process would begin with very simple steps: “you wouldn’t teach everything all at once but you would start with the most basic step. The first step was for her to take the sanitary pad when I gave it to her” (educator). The expected time frame for learning menstrual care varied significantly between girls. For example, one educator estimated a year and a half for some girls, while another parent felt that their daughter may not ever achieve this skill to full independence. In cases where interviewees felt that full independence may not be achieved, an emphasis was placed on each girl reaching their maximum potential and maintaining that level of skill: “getting to a stage where we are going to get the best out of her and we just have to maintain the skill that she has” (parent).

Educators highlighted that “there are many skills they have to learn, but obviously they have to be tailored to where they are” (educator). One school had put together a hierarchy of skills in relation to puberty ranging from “must have skills” to “good to have skills”, which helped guide educators in relation to supporting the girls: “must have skills were like very, very basic skills like being able to take your trousers up and down. And then they build up and up and up until at the very top level they would be being able to predict when you’d be on your period, being able to communicate your emotional states related to it” (educator). One educator commented that this hierarchy had put the school in a “much better position” as educators could refer to it to choose skills for girls to work on: “we looked at the specific learners and what they had in that hierarchy of skills, and what things were missing and picked targets that way” (educator).

Parents and educators identified *using a range of strategies and supports* in relation to supporting the girls through puberty, including Social Stories (to explain inappropriate touching, for example), visual strips (teaching skills in a sequence with visual guides), life size dolls (for showing girls which areas of the body are private), calendars (to predict when girls will be menstruating), vibrating watches, and researching the topic of puberty online. While there appeared to be many resources on which to draw, the heterogeneity of the girls was recognised, and it was felt to be important to identify what would be beneficial for each girl, on an individual basis. For example, one parent stated that her daughter did not relate to cartoons and suggested making non-animated videos that simply showed how to carry out menstrual care routines: “an actual video, which is not publicly available but just available for girls who are reaching puberty: you go to the toilet, this happens, you put the pad in, it’s ok” (parent). Another parent suggested the development of more technology-based resources, by “knowing what is out there for typical girls” (parent) and adapting them. It was also felt that resources should be adapted for girls with other additional needs: “people need to do a sit down and big brain storming thing about this. And then think, oh you used that for epilepsy, that’s a really good idea; we could use that for girls and periods” (parent). Many parents used the Internet, albeit with caution. For example, one parent read a suggestion to insert a tampon for her daughter, allowing her to access swimming and related activities. This parent considered this to be an inappropriate solution, as it was an invasion of her daughter’s privacy: “that’s something I wouldn’t actually ever be comfortable doing. And I sort of thought that would be almost like a violation for me to do something like that for her” (parent).

Encouragingly, parents spoke positively about their daughters’ schools in relation to supporting the girls through puberty. Most parents talked with the schools prior to their daughters reaching puberty to discuss what would be taught and when, and many educators provided parents with resources for home (e.g. Social Stories, visual strips). However, some parents and educators felt that the schools learnt through practice in this area, and as there were fewer girls than boys in such schools, they did not have as much experience in addressing puberty: “It’s not something that’s sort of a well-trodden path” (parent).

Educators highlighted the importance of caring about the girls and knowing them well, and reported their own personal experience of puberty to be a valuable resource in noticing and understanding changes relating to puberty: “because men tutors haven’t been through it, I think it would be harder for them to notice those changes” (educators). They did, however, report needing extra support. Several educators suggested that it might be beneficial to have an external person come into the school, to support them in teaching puberty-related skills: “it would be a good thing to have either a nurse, or someone who’s more experienced in broaching this subject, to help us, how to explain it to our pupils. It’s not even about feeling awkward, I don’t have any problems talking about it. It’s more, how do I say it in an appropriate way that will make them understand?” (educator). One educator who had access to such a person in her school spoke highly of them and expressed concern that they may not have access to this support in future: “if we didn’t have that specialist it would be a very, very difficult matter for me as well. And, actually, from what I’ve heard, they are cutting down their funding so we are not going to have her from next year” (educator).

Both parents and educators highlighted the importance of *open communication* in supporting the girls: “the parents were very supportive, the school was very supportive. Like there was no one who didn’t want to help, to further help the girls to be independent” (educator). Interviewees highlighted the need for a strong relationship between home and school, as both relied on each other for information and/or strategies: “you have to have an open relationship with the parents because you know, they have to tell you when the period is coming like, or else you need to track it yourself, but they were very good for telling us” (educator). There were also clear support networks within participant groups, with educators discussing specific issues with one another, and parents doing similarly: “I spoke about it with other mums here of girls, and we had chats and I found that really helpful” (parent). While the importance of open communication was emphasised throughout, it was not always well executed. Some educators reported having little contact with home, and other parents spoke of how they didn’t always know what was happening at school: “I feel like I never quite get a straight answer out of [school] or the doctors” (parent).

## Discussion

There is limited research on puberty in relation to autism, and especially in minimally verbal autistic girls with intellectual disabilities. The present study aimed to extend knowledge in this area by focusing on the support needs of this sample in relation to puberty, from the perspectives of parents and educators. Results demonstrated that while parents had concerns prior to the onset of puberty in these girls, parents and educators felt that, for most girls, experiences of puberty were positive and that they reportedly coped well with changes that they were experiencing (e.g. menstruation, breast development and developing body hair). Thematic analysis of interview data highlighted three main themes: a range of individual experiences and needs; the importance of promoting dignity and respect; and identifying ways to support these girls through puberty.

As found in previous research, in relation to both girls with intellectual disabilities (Zacharin 2009) and girls with an autism diagnosis (Cridland et al. 2014; Koller 2000), parents in the current sample expressed concerns prior to their daughters starting puberty. However, these worries subsided over time, as they realised that their daughters were coping quite well with the changes associated with puberty. Parents were also happy that, not only were their daughters’ bodies developing as expected, their daughters were responding well to these changes. These findings may help ease parental concerns prior to puberty.

Menstruation and menstrual management were a main focus of the interviews with parents and educators in this sample—an unsurprising finding, given that the majority of research on puberty tends to focus on menstrual care and hygiene (e.g., Carlson and Wilson 1994; Joshi and Joshi 2015; Klett and Turan 2012; Veazey et al. 2016). Parents and educators emphasized how menstrual management was key to promoting the girls’ dignity and respect: the less reliant on others they were, in turn, the less vulnerable they were; a positive finding, given that not all women with intellectual disabilities who may have the ability to manage their menstrual care are given the opportunity to learn to do so (Rodgers and Lipscombe 2005). Memarian and Mehrpisheh (2015) noted that most girls who can use a toilet can learn to use a sanitary pad, and this attitude was evident in all interviewees in this study. For most girls, menstrual management was a long-term goal that they were working on through a series of smaller gradual steps. These smaller steps meant that each girl had something achievable to aim for, which in turn made the experience less daunting for parents. Educators emphasised the importance of having the girls’ education on puberty individualized, so that at each stage of their learning, each girl was as independent as possible, whilst striving toward increasing this level of independence. Previous research has also emphasised the importance of step-by-step guides and strategies being made available to young autistic girls and their parents, to help with menstrual management and associated difficulties (Steward et al. 2018).

Parents and educators also highlighted that individual girls need different types of support, as what may benefit one girl may not benefit another (also see Steward et al. 2018). While the girls’ puberty-related targets were similar, there were multiple types of supports and strategies used to aid the girls in achieving these goals. Parents did, however, voice their frustrations with some of these resources, as not all were accessible for this population of girls. One such example was in relation to Social Stories, which have been previously used successfully in relation to puberty in girls on the autism spectrum (Klett and Turan 2012). Parents and educators in this study noted that, for minimally verbal autistic girls with intellectual disabilities, Social Stories were not always accessible: some girls did not understand them, and/or did not relate easily to them. While Social Stories are reported to have a small clinical effect, the research is often criticised for failing to accurately describe participants (Reynhout and Carter 2011), and may well not include those individuals who have the levels of support needs and/or limited levels of spoken communication seen in the present sample. In contrast, parents and educators spoke highly of the ‘vibrating watch’ (set to vibrate at fixed intervals as a reminder to go to the toilet, to promote menstrual hygiene) as a useful resource for these girls. Similar watches have been used to increase on-task behaviours in autistic students and students with learning disabilities (Finn et al. 2015; Legge et al. 2010) and more advanced watches (with pictures, auditory and video prompts with voice over) have been used to increase autistic individuals’ independence in relation to specific skills (e.g. cooking; Mechling et al. 2009), with students able to adjust prompt levels as they achieved increased independence over time. The possibility of adapting these more advanced watches (with pictures etc.) for puberty should be considered. Given the importance both parents and educators placed on independence, this could allow for educators and caregivers to fade their prompt levels in relation to intimate skills (i.e. toileting and puberty related skills), potentially affording the girls more dignity, respect and independence (which they stressed as being of utmost importance).

Another common concern that both parents and educators addressed regarded period pains, which were particularly worrisome given that the girls may not be able to clearly articulate their pain. While period pains are a common complaint for females going through puberty (Zeev 2006), this finding highlights the importance of parents and educators being vigilant, to try to identify this pain in girls who may be not be able to communicate their pain clearly or effectively.

Schools were seen to play a major role in supporting this group of girls through puberty. Parents reported that they spoke to schools prior to the onset of puberty, worked collaboratively in relation to target skills, and that the schools provided additional resources for use at home. While parents were generally happy with the schools in relation to puberty, educators reported that schools needed more support in this area. One educator spoke favourably about having access to a medical expert who came into the school to speak to the girls and educators about puberty. Given the literature advocating for the involvement of experts in educating about puberty (Albanese and Hooper 2007; Joshi and Joshi 2015), having a medical expert on hand to support schools would be a valuable resource. These external medical experts could also be a valuable resource for parents who do not know who to talk to in relation to puberty. It would also promote consistency (across school and home) and support more collaborative work between parents and school (see also Ruble and Nancy 2002).

Whilst this research focused on an important topic in an under-studied area, it is not without its limitations. One major limitation of this study is that the views of the girls themselves, in relation to their experience of puberty, were not considered. We had intended to interview the girls using a bespoke sort card task: presenting the girls with a number of puberty related items (using images and words) and then asking the girls to respond by placing the image on one of three response sheets (thumbs up, neutral hand and thumbs down). However, parental consent was only provided for four girls to be interviewed. In piloting the task on these four girls, this methodology showed promise in terms of how the girls engaged and responded with the puberty-related items. Yet insufficient data were collected to draw meaningful conclusions about the girls’ own views and experiences of puberty. This is an important area for future research—especially since those with limited spoken communication are all too often excluded from research.

In conclusion, this research explored the experience of puberty for autistic girls who are minimally verbal and with intellectual disabilities, through interviews with their parents and educators. Encouragingly, and despite parental concerns, puberty was found to be a generally positive experience. Parents and educators highlighted that a key priority was for each girl having as much dignity and independence as possible and—given the heterogeneity of autism—multiple ways to achieve this aim were identified. In terms of learning skills, menstrual care appeared to be critical. This often took a long time to learn, needed to be broken down into multiple steps and individualised to each girl. In terms of education around the topic of puberty, it was felt that it was important for this to be discussed between the school and parents, and again be personalised to the specific needs of each girl. While parents and educators used numerous different resources, a stronger evidence base is needed to demonstrate their effectiveness in relation to this particular population. Future research in this area should focus on identifying developmentally-sensitive ways to elicit the girls’ ‘voices’ on their own perceptions and experiences of puberty.

## Appendix

**Table 1:** Parent and educator interview schedules including key questions and question prompts

Key questions	Probe questions
*Parent interview schedule*	
Tell me about your daughter	How old is she?
	Does she have any particular strengths/challenges?
	Has [daughter] reached puberty? When was this?
Prior to [daughter] reaching puberty, was this something you had thought about?	*If yes* What types of things did you think about? Why were these things important to you? Were there any concerns/issues you had in relation to puberty, or anything in particular you were looking forward to? Why was this? Were there any resources/support you used during this time, prior to [daughter] reaching puberty? Were these helpful? If yes, why? If no, why? What other resources/support would you have liked at this time? *If no* Why wasn’t this something you considered?
Tell me about [daughter’s] experience of puberty	Since [daughter] reached puberty, have you seen any changes in [daughter’s] life? If so, what changes? Have these been positive or negative changes? Why?
	Are there any new skills [daughter] had to learn as a result of reaching puberty? If so, how did [daughter] learn these skills? Was this something she found easy or difficult? What could help support you or X with this?
	Are there any resources/support you and [daughter] used in learning these skills and managing these changes? What are your thoughts on these? If good, why? If not so good, why? What else would you have benefited from and why?
Moving forward, what (if anything) could be done to improve your daughter’s experience of puberty?	Is there anything specific you want your daughter to learn in relation to puberty?
	Can you think of any resources that aren’t available to you which could help you and your daughter in relation to her experience of puberty?
*Educator interview schedule*	
Could you tell me a little about your professional experience, and specifically your experience working with autistic girls who have reached puberty?	What is your current role?
	How long have your been working in education?
	How long have you been working with learners on the autism spectrum?
	How long you have been working with autistic girls who are non-verbal and with intellectual disabilities?
	Have you worked with these girls whilst they have been experiencing puberty?
	In what capacity have you been working with these girls?
What do you consider to be the main changes that occur at this time for girls?	How do the girls manage these changes? Are there any aspects they seem to cope well with? Are there any aspects they struggle with? What could be done to best support them?
	How do you help to manage these changes? What works well? What challenges do you face? What could support you in this regard?
	Are there any particular positive or negative changes/outcomes relating to puberty that you can think of? *(If positive, prompt negative and vice versa)*
	Were there ever any issues/concerns you had for the girl(s) relating to puberty? *If yes*: What were your concerns? Did you act on these? If yes, how? Did you feel you could manage these? Is there any further support you would have liked?
Tell me about what the girls learn and how they are supported in relation to puberty	What kinds of skills do the girls have to learn in relation to puberty? How do you teach these? How successful are the strategies? Is there anything that works particularly well? Is there anything that is more difficult?
	Can you think of any specific challenges or successes in relation to skills being learned? Could you give me an example of a skill a girl successfully learnt in relation to puberty? Can you think an example of a time where a girl had a difficult experience in relation to puberty? What do you think could have been done differently in this situation?
	How are these girls supported through puberty? Are you satisfied with this level of support? Were there any resources/support you used in order to teach these skills or teach these girls about puberty more generally? If yes What was your opinion on the resources you used? Are there any additional resources you would have liked/potentially benefitted from? Why? Are these things easy to access? If no Why not? What would you have liked?
	In relation to the parents of the girls you work with, do you feel that they are adequately supported in relation to their child’s experience of puberty? Why/why not? What could be done to further support them?
Moving forward, what do you think could be done differently to best support these girls through puberty?	Can you think of anything that could be done to help in your role, supporting these girls through puberty?
	Based on your experience, what do you think are the most important skills for these girls to learn?
	What do you think could be done differently in order to best support these girls through puberty

## Footnotes

1 Throughout this paper, we adopt identity-first (i.e., autistic person) rather than person-first (i.e., person with autism) language, in line with the preferences of many members of the autistic community (Kenny et al. 2016; Sinclair 1999).
